# Depression and Physical activity among cardiac patients undergone cardiac events: a correlational study*

**DOI:** 10.17533/udea.iee.v41n2e12.

**Published:** 2023-08-25

**Authors:** Sawroop Dhillon, Harmeet Kaur Kang

**Affiliations:** 1 RN, Ph.D. Scholar, MSN. Assistant Professor. Chitkara School of Health Sciences, Chitkara University, Punjab India; Centre for Evidence based Practice in Healthcare, Chitkara University, Punjab, India Email: sawroop.dhillon@chitkara.edu.in. Author https://orcid.org/0000-0001-8358-0378 Chitkara University Chitkara School of Health Sciences Chitkara University Punjab India sawroop.dhillon@chitkara.edu.in; 2 RN, Ph.D. Professor. Chitkara School of Health Sciences, Chitkara University, Punjab India; Centre for Evidence based Practice in Healthcare, Chitkara University, Punjab, India Email: harmeet.kaur@chitkara.edu.in https://orcid.org/0000-0002-6262-0390 Chitkara University Chitkara School of Health Sciences Chitkara University Punjab India harmeet.kaur@chitkara.edu.in

**Keywords:** depression, exercise, cardiovascular diseases, depresión, ejercicio físico, enfermedades cardiovasculares, depressão, exercício físico, doenças cardiovasculares

## Abstract

**Objective:**

To assess prevalence of depression and its relationship with physical activity among individuals who have experienced a cardiac event.

**Methods:**

This descriptive study involved 196 cardiac patients receiving treatment at selected cardiac hospitals of Punjab (India). Subjects were chosen using purposive sampling technique. After getting informed written consents from the participants the data was collected using International Physical Activity Questionnaire (IPAQ) and Beck Depression Inventory-II (BDI-II).

**Results:**

The results showed that majority (62.2%) of the cardiac patients had moderate clinical depression and 11.2% of patients had severe depression. 86.7% of the patients had low level of physical activity (<600 MET min/week). There was also a significant negative correlation between the depression and physical activity depicting the higher the physical activity, lower was the depression score and vice-versa (*p*<0.05). Moreover, study results revealed that physical activity was significantly associated with age and educational status; whereas, depression was not associated with selected demographic variables.

**Conclusion:**

The current investigation has brought to light that a vast majority of individuals suffering from cardiac issues exhibited signs of moderate to severe depressive symptoms. Additionally, the findings indicate an inverse relationship between depression and physical activity. Consequently, it is crucial for nurses to concentrate on identifying early indicators of depression and physical inactivity so that individualized care plans can be developed to enhance the overall health of cardiac patients.

## Resumen

**Objetivo:**

Evaluar la prevalencia de la depresión y su relación con la actividad física entre las personas con que han presentado un evento cardíaco.

**Métodos:**

Este estudio descriptivo involucró a 196 pacientes cardíacos que recibieron tratamiento en hospitales cardíovasculares seleccionados de Punjab (India). Los sujetos fueron elegidos utilizando la técnica de muestreo intencional. Después de obtener los consentimientos informados por escrito de los participantes, los datos se recopilaron utilizando el Cuestionario Internacional de Actividad Física (IPAQ, por sus siglas en inglés) y el Inventario de Depresión de Beck-II (BDI-II).

**Resultados:**

La mayoría (62.2%) de los pacientes cardíacos tenían depresión clínica moderada y otro 11.2% tenían depresión severa. El 86.7% de los participantes tenía bajo nivel de actividad física (<600 MET min/semana). Se encontró una correlación negativa significativa entre la depresión y la actividad física, ya que cuanto mayor era la actividad física, menor era la puntuación de depresión y viceversa (*p*<0.05). Además, los resultados del estudio revelaron que la actividad física se asoció estadísticamente con la edad y el nivel educativo; mientras que no se encontró que la depresión estuviera estadísticamente asociada con variables demográficas seleccionadas.

**Conclusión:**

El presente estudio reveló que la mayoría de los pacientes cardíacos tenían síntomas depresivos moderados o severos. Los resultados también mostraron que la depresión y la actividad física estaban inversamente correlacionadas. Por lo tanto, el enfoque en la detección de síntomas tempranos de depresión e inactividad física por parte de los enfermeros es crucial para la formulación de un plan de atención individualizado para promover el bienestar de estos pacientes cardíacos.

## Resumo

**Objetivo:**

Avaliar a prevalência de depressão e sua relação com a atividade física em cardiopatas.

**Métodos:**

Este estudo descritivo envolveu 196 pacientes cardíacos recebendo tratamento em hospitais cardíacos selecionados em Punjab, Índia. Os indivíduos foram escolhidos usando a técnica de amostragem intencional. Após obter o consentimento informado por escrito dos participantes, os dados foram coletados por meio do Questionário Internacional de Atividade Física (IPAQ) e do Inventário de Depressão de Beck-II (BDI-II).

**Resultados:**

A maioria (62.2%) dos cardiopatas apresentava depressão clínica moderada e outros 11.2% apresentavam depressão grave. 86.7% dos participantes apresentavam baixo nível de atividade física (<600 MET min/semana). Foi encontrada correlação negativa significativa entre depressão e atividade física, pois quanto maior a atividade física, menor o escore de depressão e vice-versa (*p*<0.05). Além disso, os resultados do estudo revelaram que a atividade física esteve estatisticamente associada à idade e escolaridade; enquanto a depressão não foi estatisticamente associada com variáveis demográficas selecionadas.

**Conclusão:**

O presente estudo revelou que a maioria dos cardiopatas apresentava sintomas depressivos moderados ou graves. Os resultados também mostraram que depressão e atividade física foram inversamente correlacionadas. Portanto, o foco na detecção precoce de sintomas de depressão e sedentarismo pelos enfermeiros é crucial para a formulação de um plano de cuidados individualizado para promover o bem-estar desses cardiopatas.

## Introduction

Myocardial Infarction (MI) is a cardiac emergency that requires immediate medical attention to prevent further complications.^1^ Every year approximately 7-8 million people suffer from MI.^2^ After MI, there are severe symptoms of depression with a prevalence rate of 19.8%. More than 1/3^rd^ of cardiac patients face these symptoms for more than a year.^3^^,^^4^ Studies have shown that despite different medical approaches used, the depression among cardiac patients was not fully cured^5^ because the treatment of cardiac disorders mainly focuses on physical causes rather than psychological factors.^6^ However, many studies revealed an inverse relationship between the level of depression and the type of physical activity.^7^^,^^8^ The use of exercise programs among cardiac patients has brought significant improvement in their outcomes and decreased depression.^9^ Thus, the health care team including nurses must possess an understanding of the fact that cardiac events are accompanied by depression which must be dealt simultaneously to achieve the optimum level of health. Nurses are supposed to play a key role in the management of cardiac patients and for delivering the comprehensive nursing care. They need to keep in mind the coexisting physical and mental symptoms while planning the nursing interventions. Merely observing change in behavior and detecting depression is not easy, it needs analyzing the patient daily to find mental issues in them which a nurse can do as she is in close contact with them.^10^ Hence, the study was conducted with objectives to assess the depression and physical activity among cardiac patients and to find out the relationship between depression and physical activity among cardiac patients undergone cardiac events.

## Methods

The present study used a quantitative research approach with a descriptive research design. The study's sample size was determined using a 5% type I error level and an expected 10% of proportion in the population of clinical depression in post-cardiac event patients^11^ and d=5%, resulting in a sample size of 138. However, a larger sample size was selected to prevent the loss of participants. Between September 2022 and December 2022, 196 cardiac patients undergoing treatment at designated hospitals in Punjab, India, were enrolled using purposive sampling methods. To be eligible, participants had to have experienced a cardiac event (such as myocardial infarction, coronary artery disease, stable angina, or heart failure), undergone cardiac interventional procedures (such as Coronary Artery Bypass Grafting, Valve Replacement, Percutaneous Coronary Interventions, Pacemaker, or Implanted Cardioverter Defibrillator), and be in class-I and II according to the New York Heart Association Functional Classification of Heart Failure.

The patients who had unstable angina pectoris, acute endo-myocarditis, recent phlebothrombosis, and arrhythmia were excluded. Written informed consent was taken from subjects. Thereafter, data was collected from participants during their regular visit to the concerned cardiologists using interview technique. Physical activity was measured in terms of Metabolic Equivalent of Task (MET) min/week using the International Physical Activity Questionnaire (IPAQ) as it cannot be subjectively quantified.^12^ Low physical activity was categorized as <600 MET min/week, moderate physical activity was categorized as 600-1500 MET min/week, and high physical activity was categorized as at least 1500-3000 MET min/week. Depressive symptoms were assessed using the Beck Depression Inventory-II, which has a possible score range of 0-63. A score of more than 40 indicated extreme depression.

Ethical clearance was obtained from Institution Ethics Committee (EC/NEW/INST/2020/531/CU/09). The permission was also taken from Hospital Administrators and Consultant physicians. Confidentiality of the information was maintained. IBM SPSS (version 21) was utilized for data analysis. Demographic data (such as age, gender, marital status, etc.) as well as depression and physical activity, were presented as frequency and percentages. Pearson's correlation coefficient was used to determine the correlation between depression and physical activity. Independent T-test and One-way ANOVA were utilized to assess associations. Post-hoc tests were utilized to determine pairwise comparisons of means contributing to the overall significant difference observed while computing F statistics. The level of significance for all tests was set at *p*<0.05.

## Results

In the present study, most of the patients 76 (38.8%) were in age group 51-60 years with a male preponderance 53.6% (105 out of 196), the majority [164 (83.7%)] of the cardiac patients were married, 127 (64.8%) had educational status up to the middle, 121 (61.7%) were not-working and were homemakers, 108 (55.1%) were non-vegetarians, 165 (84.2%) were taking alcohol, 170 (86.7%) were not currently smoking or consuming tobacco, 52 (26.5%) had 2 years of duration of heart disease, 77 (39.3%) patients had coronary artery disease as primary etiology, 81 (41.3%) patients cardiac history of CABG, 98 (50%) patients had CABG as therapeutic intervention done, 128 (65.3%) patients had 3 coronary vessels blocked and 116 (59.2%) patients had comorbidity. (Table 1)

**Table 1:** Frequency and Percentage distribution of the characteristics of 196 patients

Characteristics	*n*	%
Age (in years)		
30-40	10	5.1
41-50	47	24.0
51-60	76	38.8
> 60	63	32.1
Gender		
Male	105	53.6
Female	91	46.4
Marital status		
Married	164	83.7
Single	2	1.0
Widowed/ Divorced	30	15.3
Educational status		
Up to Middle	127	64.8
Up to Secondary	37	18.9
Graduation	32	16.3
Employment status		
Not-working	121	61.7
Part-time working	75	38.3
Full-time working	0	0
Occupation		
Not-working/Homemaker	121	61.7
Business/Commercial	31	15.8
Govt./Private job	44	22.4
Dietary pattern		
Vegetarian	88	44.9
Non-Vegetarian	108	55.1
Specific habits		
Alcohol	165	84.2
Smoking	26	13.3
Tobacco	26	13.3
Duration of heart disease (in years)		
1	30	15.3
2	52	26.5
3	44	22.4
4	31	15.8
5	20	10.2
6	19	9.7
Primary etiology		
Coronary	77	39.3
Hypertension	54	27.6
Cardiomyopathy	30	15.3
Mitral Regurgitation	16	8.2
Diabetes Mellitus	12	6.1
Rheumatic Heart Disease	7	3.6
Cardiac history / Diagnosis		
Myocardial infarction	30	15.3
Stroke/Transient ischemic attack	28	14.3
Coronary Artery Bypass Graft	81	41.3
Stable Angina	29	14.8
Percutaneous Coronary Interventions	28	14.3
Therapeutic intervention done		
Revascularization	36	18.4
Percutaneous Coronary Interventions	62	31.6
Coronary Artery Bypass Graft	98	50.0
Number of vessels blocked		
1	30	15.3
2	38	19.4
3	128	65.3
Co-morbidities		
Yes	116	59.2
No	80	40.8

**Table 2:** Frequency and Percentage distribution of level of depression in 196 cardiac patients

Level of depression	Possible range	n	%
Normal	1-10	0	0
Mild mood disturbance	11-16	16	8.16
Borderline clinical depression	17-20	36	18.37
Moderate depression	21-30	122	62.24
Severe depression	31-40	22	11.22
Extreme depression	41-63	0	0

The study results revealed that the majority of the cardiac patients (62.24%) had a moderate level of depression and 11.22 percentage of patients had severe depression, while 18.37 percent had borderline clinical depression. (Table 2) Additionally, the study results demonstrated that the majority (86.73%) of cardiac patients exhibited low levels of physical activity, whereas only 13.27 percent of them followed a moderate level of physical activity.

The relationship between depression and physical activity was computed using Pearson’s correlation coefficient resulted in value of -0.368 (*p*<0.001). The null hypothesis was rejected as there was a statistically significant negative and mild relationship between depression and physical activity i.e., the higher the physical activity, the lower was the depression score. 

The association between age and physical activity among cardiac patients was found to be statistically significant (*p<*0.01) and cardiac patients in the age group 30-40 years (627.500±369.783) were physically more active than other higher age group patients. (Tables 3A and 3B)

**Table 3A:** Association between Age (in years) with the level of depression and physical activity among 196 cardiac patients

Variables	Age (in years)	N	Mean (Std. Deviation)	df	F stats	***p*-value**
Level of Depression	30-40 years	10	24.400 (6.058)	4.192	2.121	0.653
	41-50 years	47	26.191 (6.787)			
	51-60 years	76	27.842 (6.661)			
	Above 60 years	63	28.619 (6.500)			
Physical Activity	30-40 years	10	627.500 (369.783)	4.192	7.468	0.009
	41-50 years	47	396.712 (270.758)			
	51-60 years	76	327.776 (205.847)			
	Above 60 years	63	303.079 (139.315)			

**Table 3B:** Post Hoc Test- Physical Activity vs. Age

Age (in years)	N	Subset for alpha = 0.05		
		1	2
Above 60 Years	63	303.0794	
51-60	76	327.7763	
41-50	47	396.7128	
30-40	10		627.5000
Sig.		0.393	1.000

From Tables 4A and 4B, it can be seen that the association between educational status and physical activity among cardiac patients was found to be statistically significant (*p*<0.001) and patients who were graduate (519.312±332.192) were physically more active than patients in other educational groups. Furthermore, the physical activity was also found to be significantly associated (*p*<0.05) with employment status, occupation, duration of heart disease, the number of vessels blocked and the presence of comorbidities. Cardiac patients who were Part-time working (337.805±205.049), were in business/commercial (442.242±343.988), who had heart disease duration of 3 years (439.590±294.400) and had co-morbidities (369.814±252.283) were physically more active. On the other hand, depression was not found to be statistically associated with selected demographic variables; age, employment status, occupation, duration of heart disease and number of vessels blocked, and presence of comorbidities.

**Table 4A:** Association between Educational Status with the level of depression and physical activity among 196 cardiac patients

Variables	Educational Status	N	Mean (Std. Deviation)	df	F Stats	***p*-value**
Level of Depression	Up to Middle	127	27.740 (6.528)	3,193	0.269	0.764
	Up to Secondary	37	27.378 (6.897)			
	Graduate	32	26.812 (6.045)			
Physical Activity	Up to Middle	127	302.755 (173.891)	3,193	13.391	<0.001
	Up to Secondary	37	374.527 (209.437)			
	Graduate	32	519.312 (332.192)			

**Table 4B:** Post Hoc Test- Physical Activity vs. Educational Status

Educational Status	N	Subset for alpha = 0.05	
		1	2
Up to middle	127	302.7559	
Up to Secondary	37	374.5270	
Graduate	32		519.3125
Sig.		.248	1.000

## Discussion

In the present study, the majority of the patients 76 (38.8%) were in age group 51-60 years with a male preponderance 53.6% (105 out of 196) and 164 (83.7%) of the cardiac patients were married. These results were supported by the cross-sectional study conducted by Sharma Dhital P *et al.*^13^ which showed that out of 168 respondents, 60.7% were male, 96.4% were married. Similarly, Fahmi I *et al* in their study titled as Relationship between Depression and Physical Activity of Myocardial Infarction Patients after Treatment revealed that of the 150 post-treatment STEMI patients, 78.7% were male.^14^ In this study, 170 (86.7%) were not currently smoking or consuming tobacco, these results are similar to a cross-sectional study conducted in 2019 in Trinidad and Tobago that smoking was not common among participants.^15^ In this study, the results revealed that the majority 77 (39.3%) of the cardiac patients had coronary artery disease as a primary etiology. This result is supported by the study conducted by Bahall^15^ in South Trinidad which showed that the most (75%) common cardiac disease was ischemic heart disease or coronary heart disease.

In the present study, 81 (41.3%) of patients had a cardiac history of CABG, 98 (50%) patients had CABG as therapeutic intervention done, and 116 (59.2%) patients had comorbidities. Similarly, a study conducted in Nepal revealed that more than two-thirds (69.1%) of the respondents had surgery as mode of treatment. Likewise, half of the respondents (50.0%) had other comorbid conditions.^13^ Physical activity is a key component in heart disease patients that is beneficial in reducing the risk of relapse.^16^ In the present study, results of the analysis showed that 170 (86.73%) of the cardiac patients had low physical activity (<600 MET min/week). The results of this study are similar to study conducted in Indonesia (2019) where 82% of respondents were at the level of mild physical activity.^14^ Similarly, the results of the study conducted by Matthias *et al* ^17^ in 2017 in Sri Lanka, where mostly (56) respondents had low physical activity. Low physical activity is a trigger for the occurrence of myocardial infarction.^17^

Many factors are related to low physical activity. A study titled as ESC Prevention of Cardiovascular Disease Programme concluded that depression was one of the dominant factors causing low physical activity.^18^ This is same as the results of present study. The results of bivariate testing found a relationship between depression and physical activity. During the state of depression, the patient becoming silent and limit their physical activity. Patients with myocardial infarction who experience depression tend to have low physical activity, and consume a lot of alcohol which is similar to the finding of the present study where 165 (84.2%) of the cardiac patients were taking alcohol.^19^ The findings of the present study showed that out of 196 patients, 122 (62.24%) had a moderate level of depression and 170 (86.73%) had low physical activity. The results are supported by the previous study reporting 82% of respondents out of 150 post-MI patients had a mild level of physical activity and 95.7% of patients experienced moderate-severe depression.^20^ The present study revealed a mild negative correlation between the level of depression and physical activity which is in consistent with the results of the previous studies showing a significant correlation between depression and physical activity.^21^^,^^22^

The selected variables-age, educational status, employment status, occupation, duration of heart disease, number of vessels blocked, and co-morbidities had a statistically significant association with the physical activity of cardiac patients. Similarly, the findings of a study showed that years of coronary heart disease (CHD) were negatively associated with maintaining regular physical activity and the patients with co-morbidities were 1.4 times more likely to maintain regular physical activity similar to the findings of the present study.^23^

In the present study, the mean level of depression was high (27.9) among patients undergone CABG. Similarly, Tully PJ and Baker RA also revealed that on an average, 15-20% of patients had major depression after CABG.^24^ The result of the study conducted by Bahall M also showed similar findings that patients who had undergone open-heart surgery intervention experienced more depression (83.3%,*p=*0.49).^15^ Our study results are helpful in planning nursing care services for patients who had undergone cardiac events and interventions. Moreover, such findings are potentially useful while assessing the need for lifestyle management, and in designing interdisciplinary care programs for the provision of comprehensive nursing care. We recommend fellow nurses to carryout research in this area for which all the aspects of health (i.e., physical, mental, social and spiritual), and their inter-relationship can be studied. 

## Limitations

Although a correlational design was appropriate for this research study, it cannot determine a causal and effect association between depression and physical activity. Furthermore, in addition to the assessment of the relationship between depression and physical activity among post-cardiac event patients, future studies could investigate other mediating and moderating variables that were not included in the present study. Another limitation is that the results regarding the assessment of depression relied solely on the participants' self-reporting of depressive symptoms, which is a subjective, multidimensional, and dynamic concept. Additionally, the study subjects were enrolled from the selected hospitals, that might affect the generalizability of findings of the study. 

## Conclusion

Despite certain limitations, the present study showed that majority of the cardiac patients who had undergone cardiac events had moderate depressive symptoms. After carrying out further investigations, it revealed that depression and physical activity inversely affect each other. The depression is a subjective, multidimensional and a dynamic concept and screening of early signs of depression and physical inactivity by the nursing staff is of utmost importance to formulate an individualized nursing care plan. We recommend to shift the focus of treatment modalities merely from physical aspects to addition of psychological aspects of health too, to help in preventing depression and improving physical activity among cardiac patients as well as to reduce healthcare costs and increase the patients’ quality of life.
